# Active Chronic Sarcoidosis is Characterized by Increased Transitional Blood B Cells, Increased IL-10-Producing Regulatory B Cells and High BAFF Levels

**DOI:** 10.1371/journal.pone.0043588

**Published:** 2012-08-22

**Authors:** Anne Saussine, Abdellatif Tazi, Séverine Feuillet, Michel Rybojad, Caroline Juillard, Anne Bergeron, Valérie Dessirier, Fatiha Bouhidel, Anne Janin, Armand Bensussan, Martine Bagot, Jean-David Bouaziz

**Affiliations:** 1 INSERM U976, Paris, France; 2 Université Paris Diderot, Sorbonne Paris Cité, Laboratory of Immunology, Dermatology & Oncology, UMR-S 976, Paris, France; 3 Service de Dermatologie, Hôpital Saint Louis, Paris, France; 4 Université Paris Diderot, Sorbonne Paris Cité; AP-HP; Service de Pneumologie, Hôpital Saint Louis, Paris, France; 5 Service d’Anatomopathologie, Hôpital Saint Louis, Paris, France; 6 Université Paris Diderot Sorbonne Paris Cité, Laboratory of Pahology, UMR-S 728, Paris, France; National Institute of Infectious Diseases, Japan

## Abstract

**Background:**

Sarcoidosis is a multisystemic disease of unknown etiology characterized by a disproportionate Th1 granulomatous immune response in the organs involved. Plasmatic hypergammaglobulinemia and B cell accumulation in granulomatous lesions suggest the possible role of humoral immune responses in the pathogenesis of sarcoidosis. The purpose of this study is to describe B cell peripheral compartment in sarcoidosis.

**Methodology/Principal Findings:**

We analyzed blood B cell subsets and BAFF levels in 33 patients with chronic sarcoidosis (active sarcoidosis n = 18; inactive sarcoidosis n = 15) and 18 healthy donors. Active chronic sarcoidosis patients had significantly less circulating memory B cells (p<0.01), more transitional (p<0.01) and increased numbers of IL-10-producing regulatory B cells (p<0.05) compared with healthy donors and patients with inactive sarcoidosis. BAFF serum levels were significantly higher in patients with active sarcoidosis (p<0.01 versus healthy donors and inactive sarcoidosis patients) and strongly correlated with serum hypergammaglobulinemia (r = 0.53, p<0.01) and angiotensin converting enzyme levels (r = 0.61, p = <0.01).

**Conclusions/Significance:**

These data show that there is an altered B cell homeostasis in active sarcoidosis and suggest BAFF antagonist drugs as potential new treatments of this disease.

## Introduction

Sarcoidosis is a multisystemic disease of unknown etiology [1] characterized by a disproportionate Th1 granulomatous immune response in the organs involved [2]–[6]. Th1 lymphocytes predominantly secrete interleukin-2 and interferon gamma, enhance macrophage tumor necrosis factor (TNF) alpha production and amplify the local cellular immune response [1], [4]. Although innate and T cell immunity play key roles in the pathogenesis of sarcoidosis [1]–[4], several arguments suggest a potential involvement of humoral immune responses in this disease. For example, active sarcoidosis has been associated with plasmatic hypergammaglobulinemia [5], B cell accumulation has been shown in pulmonary lesions [6] and a beneficial effect of anti-CD20 monoclonal antibody B cell-depleting therapy has been reported in select patients [7]–[9]. B cells are generally considered positive regulators of the immune response in inflammatory diseases [10], but recent studies have described a new subset of B cells secreting interleukin-10 (IL-10) that down-regulate immune responses: regulatory B cells (Bregs) in mice and humans [11]–[19]. B cell-activating factor from the TNF family (BAFF), also called BlyS (B lymphocyte stimulator), is a TNF superfamily member best known for its role in the survival and maturation of B cells [20]. Increased blood levels of BAFF have been found in patients with a variety of inflammatory diseases, suggesting that excessive BAFF stimulation in humans contributes to the development of these conditions [21], [22].

We analyzed B cell subsets and BAFF levels in untreated patients with active chronic sarcoidosis and compared these results with healthy donors and inactive sarcoidosis patients. Decreased memory and increased transitional and increased IL-10-producing regulatory blood B cells were characteristic of sarcoidosis patients in the active disease phase. Increased circulating BAFF levels were found in active sarcoidosis patients and correlated with serum hypergammaglobulinemia.

## Results

### Patient Characteristics

The demographic, clinical and biological characteristics of the 18 patients with active chronic sarcoidosis and the 15 patients with inactive sarcoidosis are summarized in Table 1. None of the patients in the active sarcoidosis group were on steroid therapy or immunosuppressive drugs at the time of sampling. Serum gammaglobulin levels (normal range 6.4–13.0 g/l) were significantly higher in the patients with active sarcoidosis (mean 15.2±0.8 g/l) compared with the group of patients with inactive disease (mean 11.4±0.6 g/l, p<0.05). Serum angiotensin converting enzyme (ACE) levels (normal range 8–52 UI/l) were significantly higher in the patients with active sarcoidosis (mean 90.7±13.9 UI/l) compared with the group of patients with inactive disease (mean 40.9±10.4 UI/l, p = 0.004).

**Table 1:** Demographic, clinical and biological characteristics of 18 healthy donors and 18 active and 15 inactive chronic sarcoidosis patients.

		Healthy	Active sarcoidosis	Inactive sarcoidosis
Number		18	18	15
Gender, M/F		10/8	9/9	7/8
Age, yr		41.9±3.2 (18–61)	49.1±3.2 (27–68)	49.6±2 (37–66)
Caucasian/Asian/Afrocaribbean		Not available	9/0/9	7/0/8
Lung			16 (89%)	15 (100%)
* Radiographic stage I/II/III/IV*			*2/8/1/5*	*3/6/1/5*
Skin†			10 (56%)	13 (87%)
Joints†			5 (27%)	4 (27%)
Upper respiratory tract† ^$^			6 (33%)	3 (20%)
Gastro intestinal tract†			1 (6%)	1 (7%)
Eyes†			4 (22%)	1 (7%)
Nerves†			1 (6%)	2 (13%)
Heart†			0	1 (7%)
Gammaglobulins levels (N = 6.4–13.0 g/l)			15.2±0.81 g/l	11.4±0.65 g/l*
Angiotensin Converting Enzyme levels (N = 8–52 UI/l)			90.7±13.9 UI/l	40.9±10.4 UI/l**
Treatment	None		18 (100%)	4 (27%)
	Prednisone		0	10 (67%)
	Methotrexate		0	6 (40%)
	Infliximab		0	3 (20%)

M = male, F = female, values are given as mean ± SEM,

** p<0.01 and *p<0.05 compared with active sarcoidosis,

† Visceral sarcoidosis lesions that occurred at any time of the patient’s history. Each organ defined was either clinically active at the time of inclusion in the active group or previously involved but not actively involved at the time of inclusion in the inactive group,

$ Upper respiratory tract sarcoidosis involvement was biopsy proved in 2/9 patients.

### Increased Naive and Transitional but Decreased Memory Blood B Cells in Active Chronic Sarcoidosis Patients

**Figure 1:**
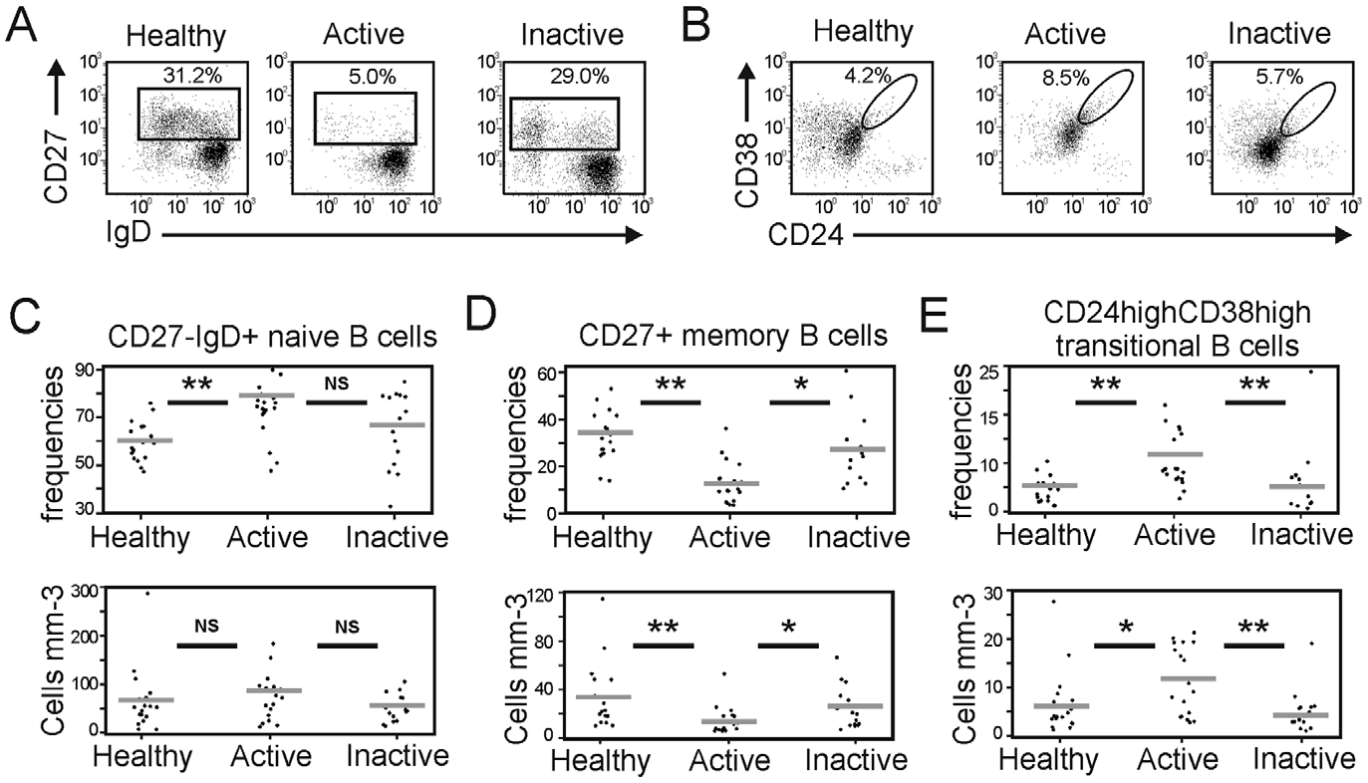
Increased naive and transitional but decreased memory blood B cells in active chronic sarcoidosis patients. (A) Representative flow cytometry dot plots of naive (CD27^−^IgD^+^) and memory (CD27^+^) B cell subsets among CD19^+^ B cells in a healthy control (healthy), active sarcoidosis patient (active) and inactive sarcoidosis patient (inactive). Numbers represent the percentage of the indicated B cell subset among CD19^+^ B cells. (B) Representative flow cytometry dot plots of the transitional (CD24^hi^CD38^hi^) B cell subset among CD19^+^ B cells in a healthy control, active sarcoidosis patient and inactive sarcoidosis patient. Numbers represent the percentage of the indicated B cell subset among CD19^+^ B cells. (C, D, E) Scatter plots showing the percentages (upper panel) and absolute numbers (lower panel) of naive (C), memory (D) and transitional (E) B cell subsets in the peripheral blood of the three subject groups as indicated. Each dot represents individual subjects, and horizontal bars represent the group means. The patients are described in Table 1. Significant differences between the means of the patient groups and healthy controls are indicated: NS, not significant; *p<0.05, **p<0.01.

To evaluate the possible changes in B cell populations in sarcoidosis patients, we compared the percentages and absolute numbers of total, naive and memory blood B cells in active and inactive sarcoidosis patients and healthy controls. Flow cytometry analysis was used to determine B cell subsets, defined as memory (CD19^+^CD27^+^) (Fig. 1A), naive (CD19^+^CD27^−^IgD^+^), and transitional blood B cells (CD19^+^CD24^high^CD38^high^) (Fig. 1B).

The percentages and absolute numbers of CD19^+^ blood B cells were similar in the 3 patient groups (healthy controls: 7.5±1.2% and 111.5±23.2/mm^3^; active sarcoidosis: 8.3±0.9% and 106.0±15.4/mm^3^; and inactive sarcoidosis: 7.3±1.0% and 84.0±10.6/mm^3^).

The inactive sarcoidosis patients had similar percentages and absolute numbers of naive B cells as the healthy controls. In contrast, the active sarcoidosis patients had a higher frequency of naive blood B cells than the healthy controls (72.1±2.7% and 59.5±1.9%, respectively; p<0.01; Fig. 1C). The inactive sarcoidosis patients had similar percentages and absolute numbers of memory B cells as the healthy controls. In contrast, the active sarcoidosis patients had decreased memory B cell percentages and absolute numbers compared with the healthy controls (13.3±2.1%, 12.9±3.0/mm^3^ and 33.7±2.5%, 35.2±6.9/mm^3^, respectively; p<0.01) and inactive sarcoidosis patients (27.8±3.7% and 24.4±5.0/mm^3^; p<0.05; Fig. 1D). The inactive sarcoidosis patients had similar percentages and absolute numbers of transitional B cells as the healthy controls. In contrast, the active sarcoidosis patients had increased transitional B cell percentages and absolute numbers compared with the healthy controls (10.8±1.2%, 11.0±1.7/mm^3^ and 5.2±0.5%, 5.9±1.5/mm^3^, respectively; p<0.01 for percentages and p<0.05 for absolute numbers) and inactive sarcoidosis patients (4.9±0.7% and 4.5±1.1/mm^3^; p<0.01; Fig. 1E).

### Identifying B Cells with Naive Phenotype in Skin Granulomas

**Figure 2:**
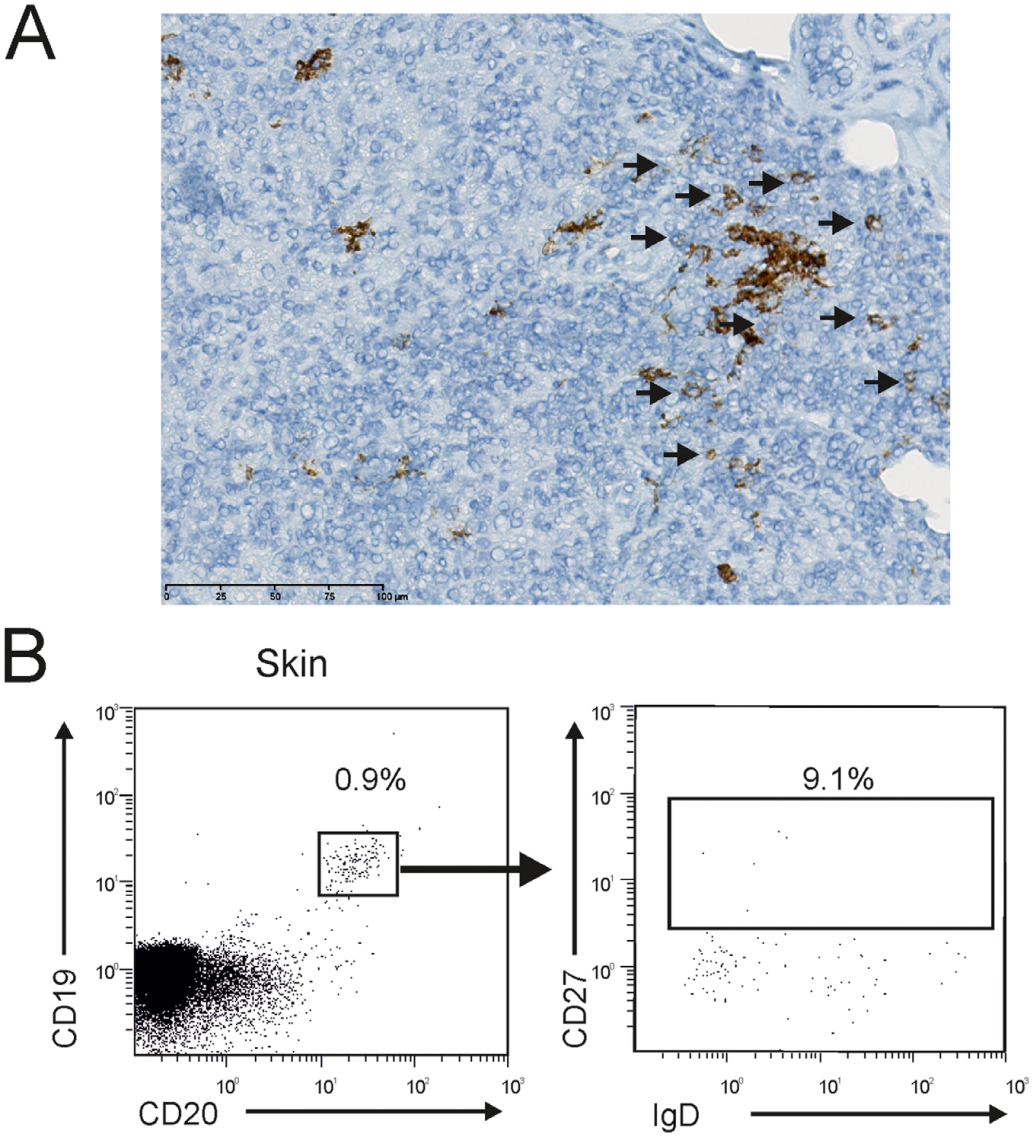
Identification of B cells with naive phenotype in skin granulomas. (A) CD20 immunohistochemical staining of a patient’s skin biopsy showing the presence of B cells in the periphery of a granuloma. B cells are indicated with a black arrow (original magnification ×400). B cells stained positive for peroxydase and had a lymphocyte-cell morphology. (B) Most of the B cells in the skin granuloma were not memory B cells but CD27^−^ B cells. CD19 and CD20 expression of the mononuclear cell infiltrate in a sarcoid skin lesion from one patient (left panel). The right panel shows the CD27 and IgD expression of skin B cells gated on the CD20^+^CD19^+^ initial population. Numbers represent the percentage of the indicated B cell subset among CD19^+^ B cells. A, B) The results are representative of three patients.

It has been hypothesized that T cell lymphopenia in sarcoidosis patients is due to T cell clustering in granulomatous lesions [23]. To assess whether decreased memory B cells in the blood was secondary to the trapping of memory B cells in tissue lesions, we determined the presence of B cells within the skin sarcoid granulomas of 3 patients. A few CD20-positive B cells were present at the periphery of the granuloma (Fig. 2A) as previously described [24]. Flow cytometry analysis of freshly isolated mononuclear cells from the skin granulomas confirmed the presence of B cells (Fig. 2B, left panel). These B cells did not belong to the memory B cell subset (CD27^−^IgD^−^ and IgD^+^) (Fig. 2B, right panel). Importantly, the collagenase digestion of blood lymphocytes did not alter the cell surface markers of B cells, including CD27 and IgD (data not shown).

### Increased Blood IL-10-producing B Cells in Patients with Active Chronic Sarcoidosis

**Figure 3:**
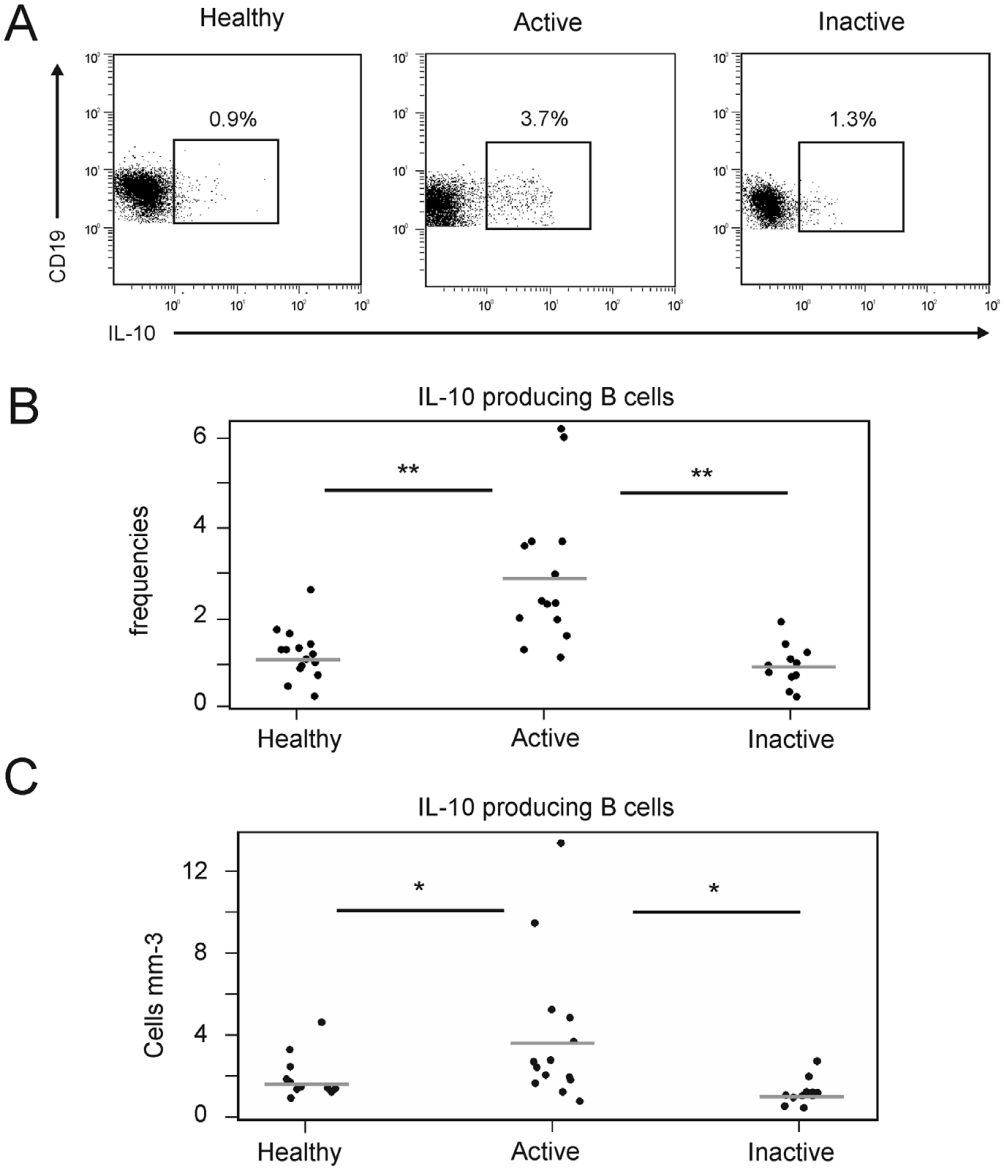
Increased blood IL-10-producing B cells in patients with active chronic sarcoidosis. (A) Representative B cell cytoplasmic IL-10 expression of 15 healthy donors (healthy), 14 active sarcoidosis patients (active) and 11 inactive sarcoidosis patients (inactive). IL-10 production by B cells was induced by 72 hours of CpG stimulation, with PMA, ionomycin and monensin being added during the final 6 hours of culture. The percentages indicate IL-10^+^ B cell frequencies among CD19^+^ B cells. (B, C) IL-10^+^ B cell frequencies (B) and IL-10^+^ B cell absolute numbers (C) as in panel A, with each dot representing individual subjects. Horizontal bars represent group means. Significant differences between the means of patient groups and healthy controls are indicated: *p<0.05; **p<0.01.

IL-10-producing regulatory B cells have been identified in mice and shown to down-regulate inflammation, making them potentially important in tolerance maintenance [25]. Several recent studies have also identified IL-10-producing regulatory B cells in humans and begun to unravel their phenotype [26]. However, the most relevant way to characterize regulatory B cells in humans is based on their ability to produce IL-10 after *in vitro* stimulation. Thus, the level of IL-10-producing B cells was evaluated in active and inactive sarcoidosis patients and healthy controls. Patients for whom stimulation could not be performed immediately at the time of blood sampling were not included in the analysis. Inactive sarcoidosis patients (n = 11) had similar percentages and absolute numbers of IL-10-producing B cells as healthy controls (n = 15). In contrast, active sarcoidosis patients (n = 14) had increased IL-10-producing B cell percentages and absolute numbers compared with healthy controls (2.9±0.4%, 3.5±0.9/mm^3^ and 1.1±0.1%, 1.6±0.3/mm^3^, respectively; p<0.01 for percentages and p<0.05 for absolute numbers) and inactive sarcoidosis patients (0.9±0.1% and 0.9±0.2/mm^3^; p<0.01; Fig. 3).

### Increased Serum BAFF Levels in Patients with Active Chronic Sarcoidosis

**Figure 4:**
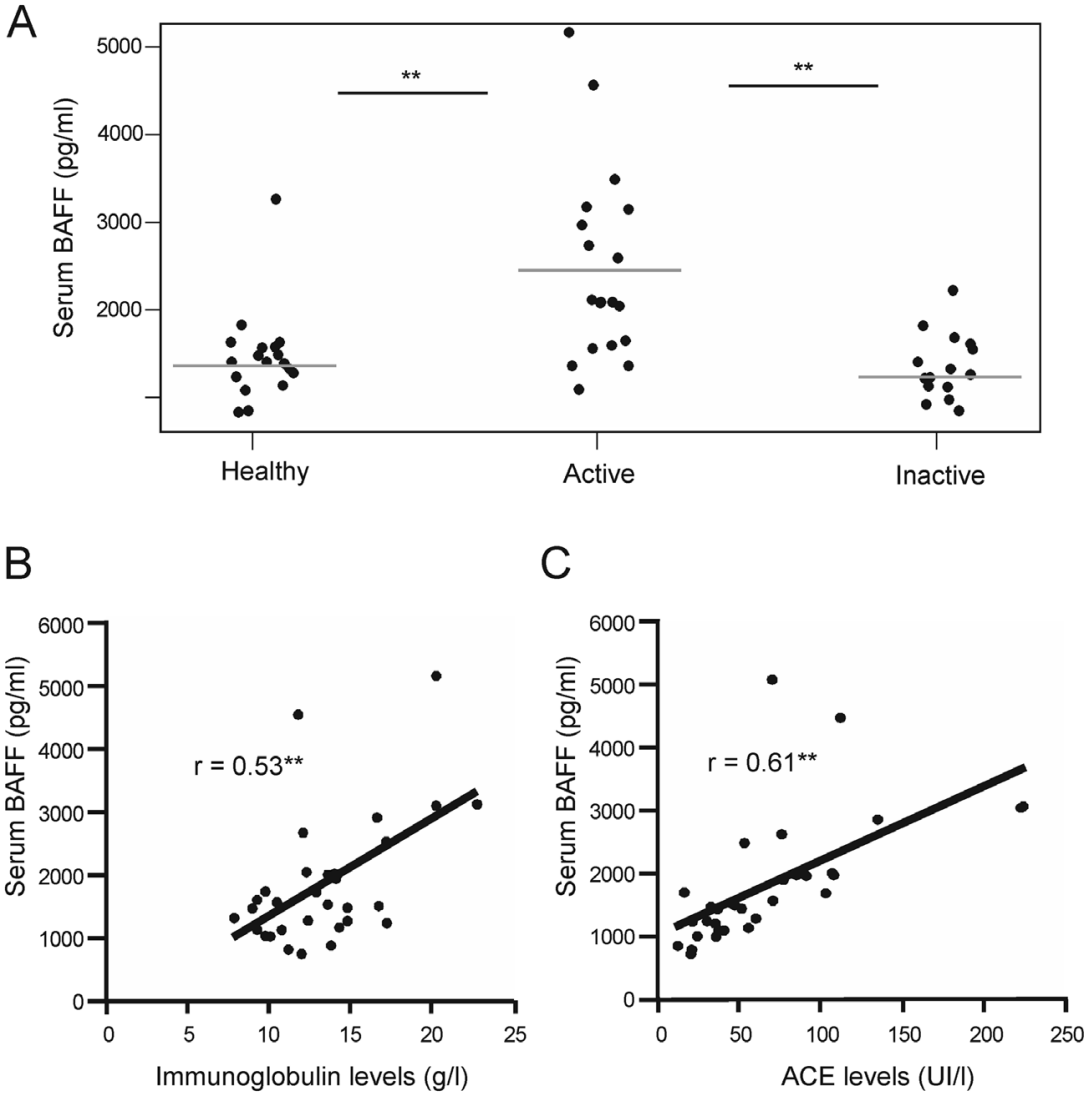
Increased serum BAFF levels in patients with active chronic sarcoidosis correlates with serum hypergammaglobulinemia. (A) Increased serum BAFF levels in active sarcoidosis patients. Scatter plots show the serum BAFF concentrations in 18 healthy donors (healthy), 18 active chronic sarcoidosis patients (active) and 15 inactive sarcoidosis patients (inactive). Each dot represents individual subjects, and horizontal bars represent the group means. Significant differences between the means of patient groups and healthy controls are indicated: **p<0.01. (B) BAFF levels correlate with serum hypergammaglobulinemia in sarcoidosis. The graph shows a linear regression analysis of BAFF levels (pg/ml) versus serum immunoglobulin levels (g/l) in sarcoidosis patients. Each dot represents individual subjects. The Pearson’s correlation coefficient is represented by “r”. The significance of the strength of the linear relationship is indicated: **p<0.01.

Increased circulating BAFF levels have been detected in several human inflammatory diseases, such as systemic lupus erythematosus (SLE), and may account for disease activity and severity [27]. We evaluated the serum BAFF levels in healthy individuals and active and inactive sarcoidosis patients. As shown in Fig. 4A, active sarcoidosis patients had significantly increased serum BAFF levels (2,343±1,079 pg/ml) compared with healthy controls (1,352±526 pg/ml; p<0.01). Inactive sarcoidosis patients had circulating BAFF levels comparable to healthy donors (1,239 pg/ml±376; NS, inactive versus healthy; p<0.01 inactive versus active). BAFF levels were strongly correlated with serum hypergammaglobulinemia in patients with sarcoidosis (r = 0.53; p = 0.0015; Fig. 4B). This correlation was not statistically significant when analyzing separately the active sarcoidosis sub-group (r = 0.43; p = 0.08). BAFF levels were also strongly correlated with serum ACE levels in patients with sarcoidosis (r = 0.61; p = 0.00022; Fig. 4C). There was a negative correlation between serum BAFF levels and memory B cell frequencies (r = 0.5198; p = 0.003) but there was not correlation between BAFF levels and others B cell subsets (transitional, IL-10 producing B cells; data not shown).

## Discussion

In active sarcoidosis, the altered blood B cell homeostasis described in the present study, i.e., decreased memory B cells, increased transitional B cells, increased IL-10-producing regulatory B cells and increased BAFF levels could be responsible for the physiopathology of the disease because these abnormalities were not found in patients with inactive sarcoidosis. The decreased memory B cell population found in active chronic sarcoidosis patients is in accordance with the results reported by Lee et al. [28]. However, their study population was heterogeneous because they included both untreated and treated patients at the time of blood collection. In our study, all the active patients were screened before any treatment to detect biological abnormalities at the peak of disease activity. In the inactive group most of the patients still received low dose of steroids or methotrexate or infliximab which does not rule out the impact of these treatments on B cell phenotypes. Our data also suggest that the decrease in memory B cells is not due to the clustering of this B cell subset in granulomatous lesions. It remains possible that some memory B cells are localized within the granulomas of other organs, including the lung bronchoalveolar fluid. An abnormal expansion of the transitional B cell subset was also noted in active sarcoidosis patients. The expansion of the transitional B cell pool has also been described in SLE patients in whom increased BAFF levels have been correlated with transitional B cell expansion [29], which was not the case in our sarcoidosis patients.

The current knowledge of IL-10-producing Bregs in humans is too preliminary to draw any definitive conclusion about their physiological relevance in human pathology. Contradictory results have been published in the literature concerning Bregs in human inflammatory diseases. First, IL-10-producing B cells may be deficient, thus contributing to disease pathogenesis. This theory is unlikely in sarcoidosis because this B cell subset is expanded. Second, IL-10-producing B cells in humans may have no effect on the initiation of inflammation but may serve as a “rheostat” to fight against inflammation once established. This theory may be the case in sarcoidosis. Consistent with this hypothesis, patients with inflammatory diseases (SLE, rheumatoid arthritis, Sjögren’s syndrome, autoimmune vesiculobullous skin disease, and multiple sclerosis) have shown significantly increased frequencies and absolute numbers of IL-10-producing B cells [15]. In active sarcoidosis, the expansion of the natural Treg cell subset may account for anergy by abolishing IL-2 production and strongly inhibiting T cell proliferation [30]. The same hypothesis may be formulated for Bregs implicated in sarcoidosis. Moreover, IL-10 (along with IL-4 and IL-13) appears to play a key role in the development of fibrosis, a characteristic feature of chronic sarcoidosis lesions [1], [31].

BAFF is a cell survival factor that binds 3 membrane receptors (TACI, BCMA, and BAFF-R/B lymphocyte stimulator receptor 3 [BR-3]) on B lymphocytes. BAFF inhibits B cell apoptosis and stimulates the differentiation of B cells into immunoglobulin-producing plasma cells [32]. BAFF is over-expressed in patients with SLE and rheumatoid arthritis in whom BAFF levels have been correlated with IgG levels and disease activity [27]. Similarly, we demonstrated that BAFF levels were selectively increased in active sarcoidosis patients and correlated with serum hypergammaglobulinemia, a well-known feature of sarcoidosis activity [5]. Belimumab is a fully human IgG1ë monoclonal antibody that binds to soluble human BAFF and inhibits its biologic activity. It has been shown to be efficacious and safe in patients with active SLE in a randomized, placebo-controlled phase 3 trial [33]. The current study on BAFF levels in sarcoidosis suggests that belimumab or other BAFF antagonist drugs may be a potential treatment of this disease in the future.

## Materials and Methods

### Patients

All patients’ blood and skin samples were obtained after written informed consent in accordance with the Declaration of Helsinki and approval by our institutional review board (CPP, University Paris 12, France). Thirty-three patients who had clinical and histological features consistent with chronic sarcoidosis were prospectively included in this study. Sarcoidosis was diagnosed according to the guidelines of the American Thoracic Society/European Respiratory Society/World Association of Sarcoidosis and other Granulomatous Disorders statement on sarcoidosis [34]. Patients with Löfgren’s syndrome were not recruited because this acute form of sarcoidosis spontaneously recovers in most cases and may have a different immune response. Patients with active sarcoidosis had a progressive disease based on clinical, radiological and lung function evaluations, as previously defined by Boudouin and du Bois [35] and the consensus conference of the third WASOG meeting [36]. The criteria used were: 1) recently developed or increasing cough or dyspnoea; and/or 2) systemic symptoms including cutaneous lesions, weakness, fever, arthralgia; and/or 3) increasing opacities on chest radiography. Evaluation of disease activity was based on the clinical assessment by the medical doctors in charge of the patient (AS, SF, AT, MR, CJ, JDB) and the results of the investigations (for example, lung function testing, slit lamp examination, electrocardiogram and echocardiogram).

All active sarcoidosis patients had a disease that required the initiation of systemic therapy, including corticosteroids, methotrexate or infliximab. Patients with inactive sarcoidosis had no clinical sign of disease activity and stable chest X-ray lung abnormalities that occurred either spontaneously or during the tapering phase of systemic treatment. Blood samples were collected from active sarcoidosis patients before any treatment (n = 18) and from patients with inactive disease (n = 15). Blood samples from age-and sex-matched control healthy donors (n = 18) were collected at the “French Blood Center” (Saint Louis Hospital, Paris, France).

### Antibodies and Immunofluorescence Analysis

Peripheral blood mononuclear cells were purified using centrifugation over a Ficoll-Hypaque density gradient, with >98% cell viability. The anti-human fluorochrome-conjugated antibodies used included CD19 (SJ25C1), CD24 (ML5), CD38 (HB7) (all from BD Bioscience), CD27 (0323, eBioscience), IgD (IgD26, Miltenyi Biotec), CD20 (B9E9, Beckmann Coulter) and isotype-matched control antibodies. Cells were analyzed using CyAn flow cytometers (Beckmann Coulter).

### Intracellular IL-10 Analysis

Intracellular IL-10 analysis was performed as previously described [11]. Briefly, cells (10^6^ cells/ml) were suspended in complete medium [RPMI 1640 containing 10% human serum, 200 mg/ml penicillin, 200 U/ml streptomycin, 4 mM L-glutamine, and 1 mM sodium pyruvate (all from Gibco)] in the presence of CpG-B 2006 (0.1 µg/ml, Tib Molbiol) in 24-well flat-bottom plates for 72 hours at 37°C. Phorbol myristate acetate (PMA) (50 ng/ml), ionomycin (1 mM) and monensin (2 mM; eBioscience) were added for the last 6 hours. After membrane staining, cells were fixed and permeabilized using Cytofix/Cytoperm (BD Pharmingen) and stained with anti-human PE-conjugated IL-10 or PE-conjugated mouse IgG1 isotype control (Miltenyi Biotech).

### Immunohistochemical Staining

Fixed and paraffin-embedded sarcoid skin biopsies were analyzed for CD20 expression (CD20 mAb, clone L26, DAKO, dilution 1/400) as previously described [37].

### Skin Mononuclear Cell Isolation

Skin samples were mechanically dissociated, treated with 1 mg/ml collagenase Ia (Sigma, Roche, 2 mg/ml, 37°C, 30 min) and filtered before flow cytometry analysis.

### ACE Activity Assay

In vitro quantitative analysis of ACE activity was measured in the serum by optical density using the Infinity™ ACE Liquid Stable Reagent enzymatic kit (Thermo Scientific) according to the manufacturer’s instructions.

### BAFF Enzyme-linked Immunosorbent Assay

Serum BAFF levels were quantified using the human BAFF/Blys Quantikine® ELISA immunoassay (R&D Systems) according to the manufacturer’s instructions. Briefly, blood was collected in a serum separator tube. Samples were allowed to clot for 30 min before centrifugation for 15 min at 1,000×g. The centrifuged serum was immediately stored in aliquots at −80°C and used in duplicate after the first thaw.

### Statistical Analysis

The data are shown as the mean (±SEM). The differences between active and inactive groups of sarcoidosis patients and healthy controls were determined using the non-parametric Mann-Whitney test. The correlation between biological parameters was determined using the Pearson correlation test. P values <0.05 were considered statistically significant.
